# Antinematode Activity of Violacein and the Role of the Insulin/IGF-1 Pathway in Controlling Violacein Sensitivity in *Caenorhabditis elegans*

**DOI:** 10.1371/journal.pone.0109201

**Published:** 2014-10-08

**Authors:** Francesco Ballestriero, Malak Daim, Anahit Penesyan, Jadranka Nappi, David Schleheck, Paolo Bazzicalupo, Elia Di Schiavi, Suhelen Egan

**Affiliations:** 1 School of Biotechnology and Biomolecular Sciences and Centre for Marine Bio-Innovation, University of New South Wales, Sydney, New South Wales, Australia; 2 Department of Chemistry and Biomolecular Sciences, Macquarie University, Sydney, New South Wales, Australia; 3 Biology Department, University of Konstanz, Konstanz, Germany; 4 Institute of Genetics and Biophysics "Adriano Buzzati Traverso", National Research Council, Naples, Italy; UMASS Medical School, United States of America

## Abstract

The purple pigment violacein is well known for its numerous biological activities including antibacterial, antiviral, antiprotozoan, and antitumor effects. In the current study we identify violacein as the antinematode agent produced by the marine bacterium *Microbulbifer* sp. D250, thereby extending the target range of this small molecule. Heterologous expression of the violacein biosynthetic pathway in *E. coli* and experiments using pure violacein demonstrated that this secondary metabolite facilitates bacterial accumulation in the nematode intestine, which is accompanied by tissue damage and apoptosis. Nematodes such as *Caenorhabditis elegans* utilise a well-defined innate immune system to defend against pathogens. Using *C. elegans* as a model we demonstrate the DAF-2/DAF-16 insulin/IGF-1 signalling (IIS) component of the innate immune pathway modulates sensitivity to violacein-mediated killing. Further analysis shows that resistance to violacein can occur due to a loss of DAF-2 function and/or an increased function of DAF-16 controlled genes involved in antimicrobial production (*spp-1*) and detoxification (*sod-3*). These data suggest that violacein is a novel candidate antinematode agent and that the IIS pathway is also involved in the defence against metabolites from non-pathogenic bacteria.

## Introduction

Parasitic nematodes are an important group of human, animal and plant pathogens representing a major threat not only to public health, but also to livestock and agricultural industries around the globe [1]. Yet heavy reliance on the few available chemotherapeutic agents has resulted in the development of nematode resistance and little progress has been made in the search for new treatments [2], [3]. Thus there is an urgent requirement for the discovery of new antinematode compounds that can be developed into chemotherapeutic drugs.

The nematode *C. elegans* is a powerful model organism used broadly across the fields of cellular biology, developmental biology and neurobiology, and more recently also as a model organism for the study of host-microbial interactions with a focus on pathogenesis and drug discovery [4], [5]. In a recent functional screen of genomic libraries of marine bacteria, a number of fosmid clones expressing high toxicity towards *C. elegans* were identified [6]. One of the highly toxic clones (designated 20G8) with a sequence-insert originating from the marine bacterium *Microbulbifer* sp. D250, expressed a violet pigment. Genetic analysis of the insert revealed that the clone 20G8 contained genes encoding for the synthesis of the indole-antibiotic violacein (*vioA-E*) [6], suggesting that this metabolite is responsible for its toxic phenotype.

Violacein is produced by several bacterial species thriving in a range of habitats such as terrestrial, marine, fresh water and glacier environments. Some of the known violacein producing organisms include *Chromobacterium violaceum* [7], *Collimonas* sp. [8], *Duganella* sp. [9], *Janthinobacterium lividum* [10], [11] and *Pseudoalteromonas* spp. [12], [13]. Violacein exhibits several biological activities with ecological relevance. Firstly, violacein has been suggested to be involved in oxidative stress resistance in *C. violaceum* [14]. Secondly, violacein producing bacteria (namely *J. lividum*) have been implicated in the natural defence of amphibians to fungal disease [15]. Finally, Matz and colleagues [12] have demonstrated that in the marine bacterium *Pseudoalteromonas tunicata*, violacein production can act as an antipredator defence mechanism against protozoan grazers. In addition to its ecological significance, violacein has also gained increasing importance for its potential medical and industrial applications. The biological activities of this compound include antioxidant, leishmanicidal, trypanocidal, antifungal, antiviral, antibacterial and antiprotozoal effects, as well as antitumoral and apoptosis-inducing activities in mammalian cancer cells (reviewed in [16]).

Although violacein has a broad range of activities, the direct molecular and cellular targets remain unknown. In addition there is limited understanding of its activity in multicellular eukaryotes and whether metazoans have the capacity to mount a defence to neutralize its activity or not. Whilst lacking adaptive immune strategies, nematodes, such as *C. elegans*, are suitable models to investigate metazoan defence as they posses a sophisticated innate immune system that protects against toxic microorganisms [17]. Key features of the nematodes defence include the conserved immune regulatory pathways, *i.e.*, the p38 mitogen activated protein kinase (MAPK) and the insulin/IGF-1 signalling (IIS) pathways [18]. In particular the IIS pathway with the gene regulators DAF-2 and DAF-16, is increasingly recognised for its important role in stress response, aging and immune homeostasis across nematodes, insects and mammals [18], [19]. Furthermore, the IIS pathway is known to play a key role in the innate immune response against different pathogen-induced stresses, including colonization [17], [20], [21] and bacterial virulence factors [22].

In *C. elegans* the binding of insulin to DAF-2 triggers a phosphorylation cascade that results in activation of PDK-1 (3-phosphoinositide-dependent kinase 1) and eventual retention of the DAF-16 transcriptional activator in the cytoplasm [23]. De-activation or loss of DAF-2 function allows DAF-16 to move to the nucleus where it enhances the expression of genes including among others *sod-3* (superoxide dismutase), *spp-1* (SaPosin-like Protein) and *lys-7*, which are involved in detoxification, antimicrobial peptide expression and antimicrobial lysozyme production, respectively [24], [25], [26]. Moreover recent data indicates that the canonical IIS signalling diverges at PDK-1 into a second arm of the pathway mediated by the protein WWP-1 (WW domain protein 1) [22]. In the present study, we hypothesised that *C. elegans* makes use of this immune response pathway not only in the situation of infection by pathogenic organisms, but also to neutralize the effect of toxic bacterial secondary metabolites, such as violacein that originate from non-pathogens. Furthermore studying the mechanisms in which *C. elegans* mediates resistance to bacterial metabolites may shed further light into their molecular/cellular targets. To address this hypothesis, we first confirm that violacein is responsible for the toxic activity against *C. elegans* in clone 20G8 and its parental strain *Microbulbifer* sp. D250. We further show that the expression of enzymes that synthesize violacein in *E. coli* facilitates bacterial accumulation in the host intestine and induces apoptosis in the nematode. Finally we demonstrate that the IIS immune pathway modulates *C. elegans* sensitivity to violacein toxicity, most likely via the control of genes involved in detoxification and antimicrobial production.

## Materials and Methods

### Strains and culture conditions

All bacterial strains and vectors used in this study are listed in Table 1. Bacteria were grown in Luria broth (LB10), nematode growth medium (NGM) [27] or marine broth (Difco Laboratories, Maryland) [28] as indicated, and stored in 30% (v/v) glycerol at −80°C. Solid medium was prepared by the addition of 19 g of agar (Oxoid, Australia) per litre of culture fluid. All strains were grown at 25°C. Where required (see Table 1), chloramphenicol (12.5 µg/ml), kanamycin (100 µg/ml), and L-arabinose (0.02%, w/v) were added to the media. *C. elegans* strains (listed in Table 2) were maintained at 20°C on NGM agar plates spread with *E. coli* OP50 as a food source [29], [30]. *C. elegans* strains were stored in glycerol (70:30 vol/vol) at −80°C [30].

**Table 1:** Bacterial strains and vectors used in this study.

Strain/Vector	Relevant characteristic or genotype	Source or reference
*E. coli EPI300-T1^R^*	F-*mcr*A Δ(*mrrhsd*RMS*mcr*BC) φ80d*lac*ZΔM15Δ*lac*X 74 *rec*A1 *end*A1 *ara*D139 Δ(*ara*, *leu*) 7697*gal*U *gal*K λ- *rps*L *nup*G *trf*A *ton*A *dhfr*	Epicentre
*E. coli* 20G8	Fosmid 20G8 cloned in *EPI300-T1^R^*; Cm^r^	This study
*E. coli* 20G8*vioA^-^*	Fosmid 20G8 mutated in *vioA* gene and cloned in *EPI300-T1^R^*; Cm^r^, Kan^r^	This study
*E. coli* 20G8*vioB^-^*	Fosmid 20G8 mutated in *vioB* gene and cloned in *EPI300-T1^R^*; Cm^r^, Kan^r^	This study
*E. coli* 20G8*vioC^-^*	Fosmid 20G8 mutated in *vioC* gene and cloned in *EPI300-T1^R^*; Cm^r^, Kan^r^	This study
*E. coli* 20G8*vioD* ^-^	Fosmid 20G8 mutated in *vioD* gene and cloned in *EPI300-T1^R^*; Cm^r^, Kan^r^	This study
*E. coli* OP50	Uracil auxotroph	[29]
*Microbulbifer* sp. D250	Wild type strain	[62]
*Microbulbifer* sp. D250 dv2	D250 strain mutated in *vioB* gene; Kan^r^	This study
pLof/Tn10 KM	Mini-Tn10 (Kan^r^); Amp^ r^	[34]
pCC1FOS^a^	Fosmid backbone for genomic library; Cm^r^	Epicentre

a Copy number inducible by arabinose.

**Table 2:** *C. elegans* strains used in this study.

Strain name	Genotype/allele designation	Relevant characteristics	Source or reference
N2 Bristol	*C. elegans* wild isolate	Wild type isolate	CGC^a^
CU1546	*smIs34*	ced-1p::ced-1::GFP + rol-6(su1006)	CGC^a^
CB1370	*daf-2*(*e1370*) III	Mutated in the insulin-like receptor DAF-2. Temperature sensitive dauer constitutive	CGC^a^
IU10	*daf-16*(*mgDf4*7) I; *rrf-3*(*pk1426*) II	Mutated in the FOXO-family transcription factor DAF-16	CGC^a^
TJ356	*zIs356* IV	Integrated DAF-16::GFP roller strain. Daf-c, Rol, fluorescent DAF-16::GFP. Overexpression of DAF-16	CGC^a^
JT9609	*pdk-1 (Sa680)* x	Mutation in the gene encoding for 3-phosphoinositide-dependent protein kinase	CGC^a^
RB1178	*wwp-1(ok1102) I.*	Mutation in the gene encoding for the WW domain protein 1	CGC^ab^
TM127	*daf-2*(*e1370*) III; *sod-3*(*sj134*) X	Double mutant in the insulin-like receptor DAF-2 and in the superoxide dismutase SOD-3	CGC^a^
MQ876	*daf-2*(*e1370*) III; *lys-7*(*ok1384*) V	Double mutant in the insulin-like receptor DAF-2 and in the putative antimicrobial lysozyme LYS-7	CGC^a^
MQ513	*daf-2*(*e1370*) III; *spp-1*(*ok2703*) III	Double mutant in the insulin-like receptor DAF-2 and in the antimicrobial peptide caenopore SPP-1	CGC^a^
GA186	*sod-3*(*tm760*) X	Mutated in the iron/manganese superoxide dismutase SOD-3	CGC^a^
RB1286	*lys-7*(*ok1384*) V	Mutated in the putative antimicrobial lysozyme LYS-7	CGC^a^
RB2045	*spp-1*(*ok2703*) III	Mutated in the antimicrobial peptide caenopore SPP-1	CGC^a^

a *Caenorhabditis* Genetics Center, the University of Minnesota.

b *C. elegans* Gene Knockout Project http://www.celeganskoconsortium.omrf.org.

### Fosmid analysis and transposon mutant library screening

A transposon mutant library of the antinematode fosmid clone 20G8 was generated using an *in vitro* transposon mutagenesis kit (EZ-Tn5 insertion kit; Epicentre) following the manufacturers' instructions. The DNA fosmid sequence for clone 20G8 is available from the National Center for Biotechnology Information (NCBI) public database (GenBank) via accession number JX523957. The subsequent library of 96 *E. coli* transposon mutants was replicated on LB10 Omnitray plates (Nunc, Denmark), and screened for loss of toxic activity towards *C. elegans* as previously described [31]. Clones that were partially or totally grazed by the nematodes were chosen for further characterization in the nematode killing assay (below). The disrupted genes were identified by outward sequencing from the transposon using the KAN-2 forward and reverse primers (Epicentre) (KAN-2 Forward Primer 5' ACCTACAACAAAGCTCTCATCAACC 3', KAN-2 Reverse Primer 5' GCAATGTAACATCAGAGATTTTGAG 3') and sequences were subjected to BLAST analysis [32].

### Purification and identification of violacein as the antinematode agent produced by *Microbulbifer* sp. D250

Violacein was purified from an overnight culture of *Microbulbifer* sp. D250, cells were collected by centrifugation and the cell pellets repeatedly extracted with 100% methanol. The resulting (pooled) crude extract was applied to a C18 solid phase extraction column (pre-packed C18 columns, 10 g, Alltech); after several washing steps with methanol:water (20 to 60% methanol), the violacein was eluted with 100% methanol. The violacein fraction from the solid phase extraction was further purified by preparative high performance liquid chromatography (HPLC), when using a Prodigy ODS3 column (Phenomenex, 150×4.6 mm, 5 µm particle size) and a methanol gradient from 0–100% methanol.

Liquid chromatography electrospray ionisation ion-trap mass spectrometry (LC-MS/MS) was employed to confirm that the purified purple pigment (see above) is violacein. Briefly, the LC-MS/MS was performed on a LCQ Deca SP Iontrap-MS/MS system (Thermo Finnigan). Up to 20 µl of the extract were loaded on a Nucleosil C18 column (125×3 mm, 5 µm particle size, Macherey-Nagel, Germany). The mobile phases used were (A) water acidified with 0.1% formic acid and (B) acetonitrile, at a flow rate of 0.2 ml/min. The gradient program was started at 20% B, and after 3 minutes, increased to 100% B over 13 min, and maintained at 100% B. For the MS conditions, the electrospray voltage was -5kV with a current of 12 µA; the sheath gas flow rate was 34l/min; the capillary was maintained at 275°C. For the MS/MS fragmentation, the mass width for isolation of precursor ions was 1.0Da, and the relative collision energy set at 40%. The MS chromatograms were recorded in the positive ion mode. The purified purple pigment eluted at 13.9 min, with an absorption scan corresponding to violacein as observed by HPLC-diode array detection (maxima at 260, 378 and 570 nm), and this peak in the MS exhibited a protonated molecule ([M+H]^+^) corresponding to violacein (MW 343Da; mass of the observed [M+H]^+^ ion, 344 Da); the MS/MS fragmentation pattern of this [M+H]^+^ ion also corresponded to violacein, with ions observed at (% basepeak) 344 (64), 326 (45), 316 (100), 301 (49), 299 (24), 273 (5), 251 (20), 211 (4), 183 (2), 158 (1) and 132 (1) Da (e.g, due to loss of water, elimination of CO and nitrogen species, and cleavages at the rings).

### Transposon mutagenesis of *Microbulbifer* sp. D250

To generate mutants of strain D250 that are unable to produce violacein, a random transposon mutagenesis was performed using the Tn-10-Km^R^ mini-transposon systems as described previously [33]. Briefly, a spontaneous streptomycin (Sm) resistant mutant of *Microbulbifer* sp. D250 was generated (D250-Sm^R^) and used as the recipient. *E. coli* containing the Tn10 based delivery plasmid pLOF/Km, encoding a kanamycin (Km) resistance gene marker [34], was used as a transposon donor. Donor cells were conjugated with Sm resistant recipient cells of isolate D250 (D250-Sm^R^) on filter discs in 1∶1 ratio and incubated for 12 hours at 30°C. The conjugation mix was resuspended in marine broth and serial dilutions of this mixture were spread on, marine agar medium supplemented with 200 µg/ml Sm and 100 µg/ml Km. Transposon mutants were allowed to grow at room temperature for 48 to 72 hours and visually screened for the loss of purple pigmentation; the selected transposon mutants and wild type cells were extracted with methanol (as described above) and loss of pigmentation in the mutants confirmed by measuring the absence of absorbance at 575 nm. The DNA flanking the transposon insertion in the relevant mutants was sequenced using a “pan-handle” method as described previously [33].

### Nematode killing assay

*E. coli* clones were pre-grown overnight at 37°C in LB10, and 10 µl of the cultures were spread onto 3.5 cm diameter LB10 agar plates supplemented with selective antibiotic and L-arabinose as required, followed by incubation at 25°C for four days. L4-stage nematodes were added to the bacterial lawns (30 to 40 per plate), incubated at 20°C, and scored for live and dead nematodes every 24 hours for 20 days. In order to avoid multiple generations of nematodes on the same plate, which may have lead to errors when scoring, the nematodes where transferred to a fresh plate each day. A random non-toxic *E. coli* clone was used as a negative control under the same conditions. A nematode was considered dead when it failed to respond to touch. Since the *C. elegans daf-2* and *pdk-1* mutants are temperature sensitive (at 20°C 15% of the population enter in the resistant dauer stage), all the *daf-2* and *pdk-1* mutant assays were carried out at 15°C. Control *C. elegans* strains were also tested at 15°C in order to avoid temperature bias in nematode's life span. Each assay was carried out in independent triplicate plates.

Since marine agar does not support *C. elegans* growth due to its high osmolarity, the standard nematode killing assay had to be modified in order to assess the toxicity of the marine isolates (*i.e.*, strain D250 and violacein deficient mutant dV2). The marine bacteria were pre-grown overnight at 20°C in liquid marine broth, and 10 µL of the cultures were spread onto 2 cm paper filters (0.22 µm, Millipore, Maryland) and the filters placed on 3.5 cm marine agar plates supplemented with selective antibiotics as required, followed by incubation at 20°C for four days. On day four, the paper filters were removed from marine agar plates and placed onto 3.5 cm LB10 agar plates, to which the L4-stage nematodes were added (30 to 40 per plate). *p* values were calculated on the pooled data of all of the experiments done in each set by using the log- rank (Mantel–Cox) method [35], [36] with the Prism software version 6.0c (GraphPad Software, La Jolla, CA, USA). A *p*<0.05 was considered significant.

### Violacein dose response assay

In order to estimate the dose response of *C. elegans* towards violacein, a nematode killing assay was carried out using 96 well agar plates as previously described [31]. *E. coli* clone 20G8 mutated in *vioA* gene (hereafter referred to as 20G8*vioA^−^* mutant, see Table 1) was pre-grown overnight in LB10 at 37°C and inoculated (2 µL) to each LB10 agar well supplemented with selective antibiotic and L-arabinose as required. Plates were thereafter incubated at 25°C for four days to grow the bacterial lawns, and then each 5 µL of the purified violacein preparation (in methanol, see above) was added at various concentrations to the side of the bacterial lawn and the methanol was allowed to evaporate before the violacein was gently mixed with the lawn by the addition of 10 µl of sterile water. *E. coli* mutant clone 20G8*vioA* ^−^ alone, and pure methanol added to a 20G8*vioA* ^−^ lawn, were used as negative controls. Methanol was allowed to evaporate before L4-stage nematodes were added (5–15 per well). The lethal concentration at which 50% of the nematodes were killed (LC_50_) was calculated by inference with the Prism software version 6.0c (GraphPad Software, La Jolla, CA, USA). The assays were carried out in independent triplicate plates. In order to determine if there was a significant difference in the survival of nematodes, a Students *t*-test was performed on the number of surviving nematodes at day seven. A *p*<0.05 was considered significant.

### Heat killing of bacterial strains

Bacterial lawns pre-grown on agar plates were heat killed at 65°C for one hour, and the plates cooled to 20°C before adding nematodes. In order to confirm that heat killed bacteria were dead, samples of the bacterial lawns were streaked on LB10 agar plates before the nematode killing assay, and 20 days after the start of the incubation with nematodes. These plates were each incubated at 37°C for 24 to 48 hours and subsequently checked for an absence of bacterial colonies.

### Microscopy

Overnight liquid cultures of each bacterial strain were spread on 3.5 cm LB10 agar plates and grown for four days. Nematode strains were exposed to bacteria for 24 hours and up to four days, depending on the assay, and placed on a microscope slide that had been immersed in 0.1 M sodium azide solution. Nematodes were examined under an Olympus DP70 digital camera system (Japan) with differential interference contrast (DIC) microscope optics. In order to detect the GFP signal indicative of an apoptosis induction, the CED-1::GFP reporter nematodes (strain CU546, Table 2) were exposed to a bacterial lawn of the 20G8 clone and 20G8*vioA* ^−^ and 20G8*vioC* ^−^ mutants for 24 hours. Nematodes were visualized under epifluorescence microscopy, and the percentage of nematodes with a GFP signal calculated for each treatment.

## Results

### Violacein is a toxic metabolite that mediates antagonistic interactions between the bacterium *Microbulbifer* sp. D250 and *C. elegans*

**Figure 1:**
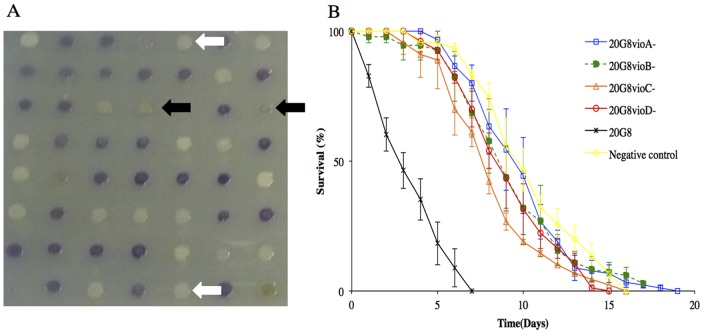
Characterization of the transposon mutant library of violacein producing clone 20G8. (A) Several mutants exhibit changes in violet pigmentation typical of the presence of violacein: 20G8*vioA* ^−^ and 20G8*vioB* ^−^ mutants have lost pigmentation (white arrows). 20G8*vioC^−^* and 20G8*vioD^−^* mutants express green-grey pigmentation (black arrows). (B) Nematode killing assay (N2 animals vs bacterial strains 20G8 clone and 20G8*vioABCD* ^−^ mutants). Survival kinetics of nematodes fed with either the 20G8 clone or the 20G8*vioABCD* ^−^ mutants deficient in violacein production. The killing phenotype of the four mutant clones is significantly reduced (*p*<0.0001) when compared to the wild type clone 20G8. A randomly chosen clone from the library with no activity is used as a negative control. Each data point represents means ± the standard error of three replicate plates. *p* values were calculated on the pooled data of all of the plates in each experiment by using the log-rank (Mantel–Cox) method.

Screening of a random transposon mutant library of the fosmid clone 20G8 for the loss of toxic activity towards *C. elegans* resulted in four mutant clones (Tables 1 and 3 and Figure 1). The nematode killing assay demonstrated that the killing phenotype of the four mutant clones was significantly reduced (*p*<0.0001) when compared to the wild type clone 20G8 (Figure 1). The activity of mutant 20G8*VioA* ^−^ was similar to the negative control (*p* = 0.803) and was therefore considered non-toxic for *C. elegans*. Sequencing of the four non-active mutants revealed that in all cases transposons had inserted in open reading frames with high sequence identity to the *vioABCDE* gene cluster of *C. violaceum*, which has previously been shown to be involved in the synthesis of violacein [37] (Table 3). Specifically, two mutants that had lost the violet pigmentation, had insertions in the *vioA* and *vioB* genes, and the two clones that expressed a grey pigmentation typical of violacein precursors, were mutated in the *vioC* and *vioD* genes (Table 3 and Figure 1) [37].

**Table 3:** Summary of the effects of violacein-producing clone 20G8 and its violacein deficient mutants on *C. elegans*.

Compound predicted to be expressed by the mutant^a^	L-tryptophan	prodeoxyviolacein	proviolacein	violacein
Proposed gene function involved in the biosynthesis^a^^,^ b	*vioAB*: tryptophan 2-monooxygenase	*vioD*: hydroxylase	*vioC*: monooxygenases	N/A
Mutant/clone name and NCBI accession number	20G8*vioA* ^−^(AFT64169) 20G8*vioB* ^−^(AFT64168)	20G8*vioD^−^* (AFT64166)	20G8*vioC^−^* (AFT64167)	20G8 (JX523957)
BLASTp analysis^c^	VioA tryptophan 2-monooxygenase; VioB polyketide synthase	VioD hydroxylase	VioC monooxygenase	N/A
Nematode survival (*p* values)^d^	*p*<0.0001	*p*<0.0001	*p*<0.0001	N/A
Colonization^e^	0%	N/A	0%	76%±1.6
Apoptosis^f^	0%	N/A	0%	70.3%±5.7
Clone pigmentation	white	grey	grey	violet

a According to [37], [45].

b Genes were ordered based on enzymatic activity in violacein biosynthesis and not on the locus position within the cluster [37].

c Only hits with 100% query coverage, 100% identity and E value of 0 were considered.

d *p* values of the survival of nematodes exposed to violacein mutants compared to the wild type clone 20G8 are reported.

e Percentage of alive nematodes colonized after four days.

f Percentage of CU1546 *C. elegans* strain displaying GFP signal indicative of apoptosis induction. All studies were carried out in *C. elegans* strain N2 except for apoptosis studies where CU1546 strain was employed. Each data represents means ± the standard error of three replicates. N/A  =  not applicable.

**Figure 2:**
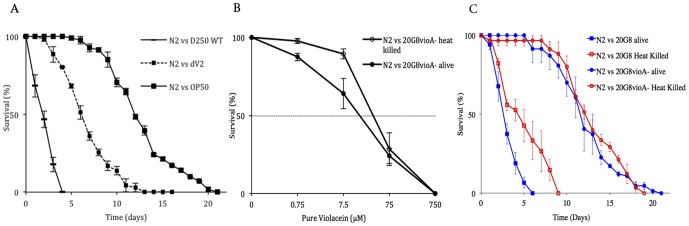
Nematode killing assay and dose response assay (N2 animal vs the alive and heat killed 20G8 clone and D250 strains). (A) Killing kinetics of *Microbulbifer* sp. D250 and dV2 mutant deficient in violacein production. Negative control OP50 is a non-pathogenic strain of *E. coli*. (B) Dose response of *C. elegans* to pure violacein added to 20G8*vioA^−^* mutant bacteria alive and heat killed. Each point on the graph represents the average survival of worms after seven days exposure to violacein (C) Kinetics of nematode killing when nematodes are fed either the live or heat killed 20G8 clone or 20G8*vioA^−^* mutant. Each data point represents means ± the standard error of three replicate plates. *p* values were calculated on the pooled data of all of the plates in each experiment by using the log-rank (Mantel–Cox) method.

To further support the involvement of violacein in nematode toxicity, *Microbulbifer* sp. D250, the parent organism for the fosmid clone 20G8, was shown to rapidly kill *C. elegans* (*p*<0.0001) when compared to negative control OP50 (Figure 2A). In contrast, the nematode's life span was significantly improved when exposed to a violacein deficient mutant dV2 (*p*<0.0001, Figure 2A). Sequencing of the transposon insertion site in strain dV2 supported the loss of violacein production, by demonstrating that the homologue to the violacein biosynthesis gene *vioB* (GenBank AFT64168) had been disrupted in the dV2 mutant. Notably, survival of the nematodes fed with the dV2 mutant strain was significantly reduced (*p*<0.0001) compared to the negative control using *E. coli* OP50 (see Figure 2A), which might indicate that other antinematode activities are also present in this bacterium. However, the involvement of violacein in toxicity towards *C. elegans* was further confirmed by the direct chemical identification of the purified violet pigment produced by *Microbulbifer* sp. D250 wild type strain. The pigment was extracted from cultures, purified and analysed by liquid chromatography-mass spectrometry (LC-MS/MS). The purified purple pigment eluted as one peak in the liquid chromatography, and this peak represented violacein, as was identified by the matching mass of the protonated molecule ([M+H]^+^) and its characteristic MS/MS fragmentation pattern (see Material and Methods).

Finally, we used pure violacein directly in the toxicity assays (see Material and Methods). The 50% survival (LC_50_) of nematodes exposed to violacein falls between the range of 7.5 µM and 75 µM of pure violacein (LC_50_ = 31.13 µM calculated from Figure 2B), when added to a viable bacterial lawn of the violacein non-producing mutant 20G8*vioA* ^−^ (Figure 2B). Interestingly, the survival of nematodes improved when violacein preparations were added to lawns of heat killed 20G8*vioA* ^−^ bacteria compared to violacein added to lawns of viable 20G8*vioA* ^−^ cells (*i.e. p* = 0.001 and *p* = 0.043 for nematodes exposed to 0.75 µM and 7.5 µM of pure violacein, respectively).

### Bacterial accumulation in the nematode intestine is involved in the killing activity of the violacein-producing *E. coli* clone

**Figure 3:**
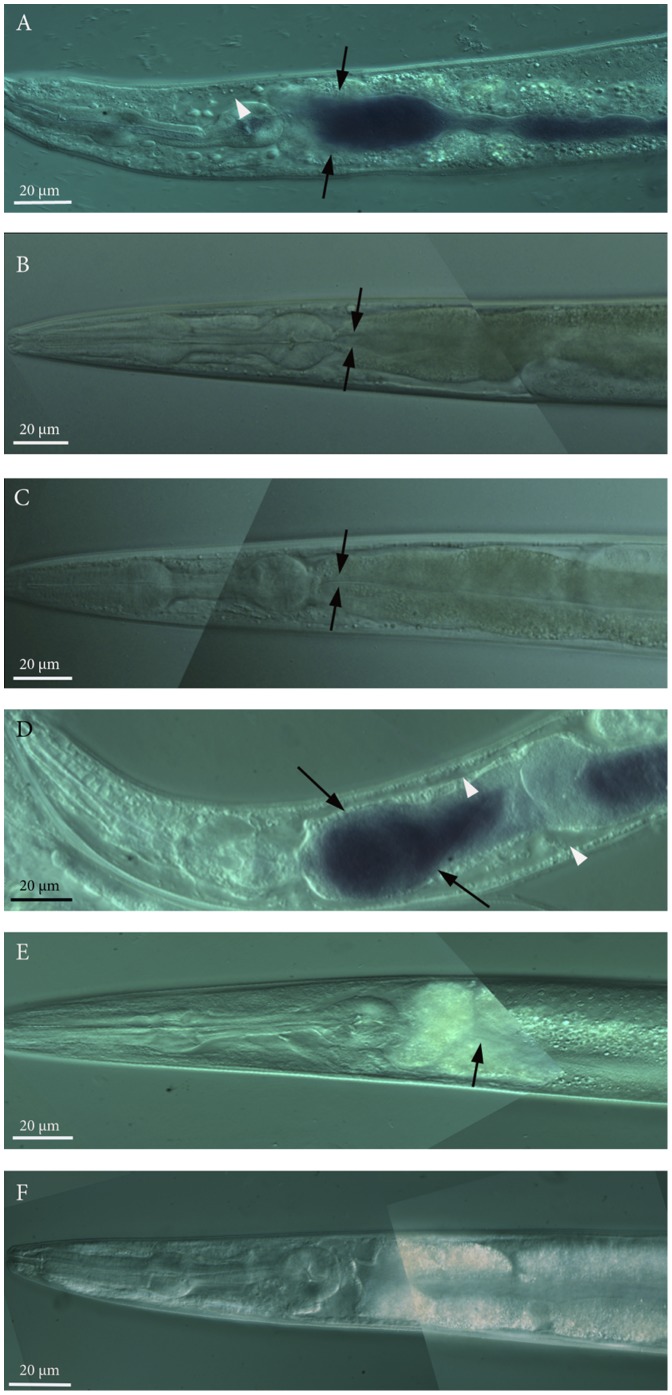
Visualization of bacterial accumulation in the nematode intestine by the *E. coli* clones (wild type animals, the *daf-2* and *daf-16* null mutant animals and the DAF-16 overexpressing nematodes vs the *E. coli* clone 20G8 and 20G8*vioA^−^* and 20G8*vioC^−^* mutant clones). All images present *C. elegans* anterior to the left and show the pharynx and first part of the intestinal lumen by differential interference contrast (DIC) microscopy. Accumulation by 20G8 cells in the nematodes intestine (arrows in panels A and D) and extensive enlargement of extracellular regions (white arrowheads panels A and D) in N2 wild type (panel A) and *daf-16* mutant (panel D) animals. No change in phenotype was observed in N2 nematodes fed with violacein deficient mutant clones 20G8*vioA^−^* (negative control-panel B) and 20G8*vioC^−^* (panel C). Similarly, no bacterial cells or enlargement of extracellular regions were detected in *daf-2* and DAF-16 over-expressing mutant nematodes exposed to the violacein producing clone 20G8 (arrows in panel E and F respectively). Each panel was assembled from multiple photomicrographs taken with the same magnification and same acquisition settings.

Given that the presence of live bacteria contributed to the decrease in the survival of nematodes in the presence of violacein (see above), we looked for evidence of bacterial accumulation in the nematodes intestine. The gut of 76% (n = 47) of the nematodes exposed to violacein-producing *E. coli* clone 20G8 had an accumulation of bacterial cells expressing a violet pigmentation in their intestine (Figure 3A). In contrast, nematodes exposed for four days to the violacein deficient mutants 20G8*vioA^−^* (n = 42) and 20G8*vioC^−^* (n = 57) showed no accumulation of bacteria in the intestinal lumen (Figure 3B and C). Microscopic analysis further showed tissue damage with enlargement of the intestinal lumen and enlargement of extracellular regions in nematodes fed with the 20G8 clone (arrowheads in Figure 3A). These data suggest that accumulation of bacteria in the intestinal lumen is one factor in the killing activity of clone 20G8. In order to further assess this, a nematode killing assay was performed using live and heat killed 20G8 cells. Heat inactivation of bacterial cells showed that nematode survival significantly increases (*p*<0.0001) in the presence of heat killed 20G8 cells compared to viable 20G8 bacteria (Figure 2C). In contrast, there was no significant effect (*p*>0.05) of heat killing on the survival of nematodes exposed to the violacein deficent mutant 20G8*vioA^−^* (Figure 2C). In order to confirm that bacterial cells were successfully inactivated by heat, they were streaked on LB10 agar plates and monitored for growth. Bacterial growth was not detected confirming that the heat treatment was sufficient to kill the bacteria. Together these data suggest that the toxic effect of violacein is significantly increased by the presence of live violacein-producing bacteria that accumulate in the intestine.

### Violacein-producing bacteria induce apoptosis in *C. elegans*

**Figure 4:**
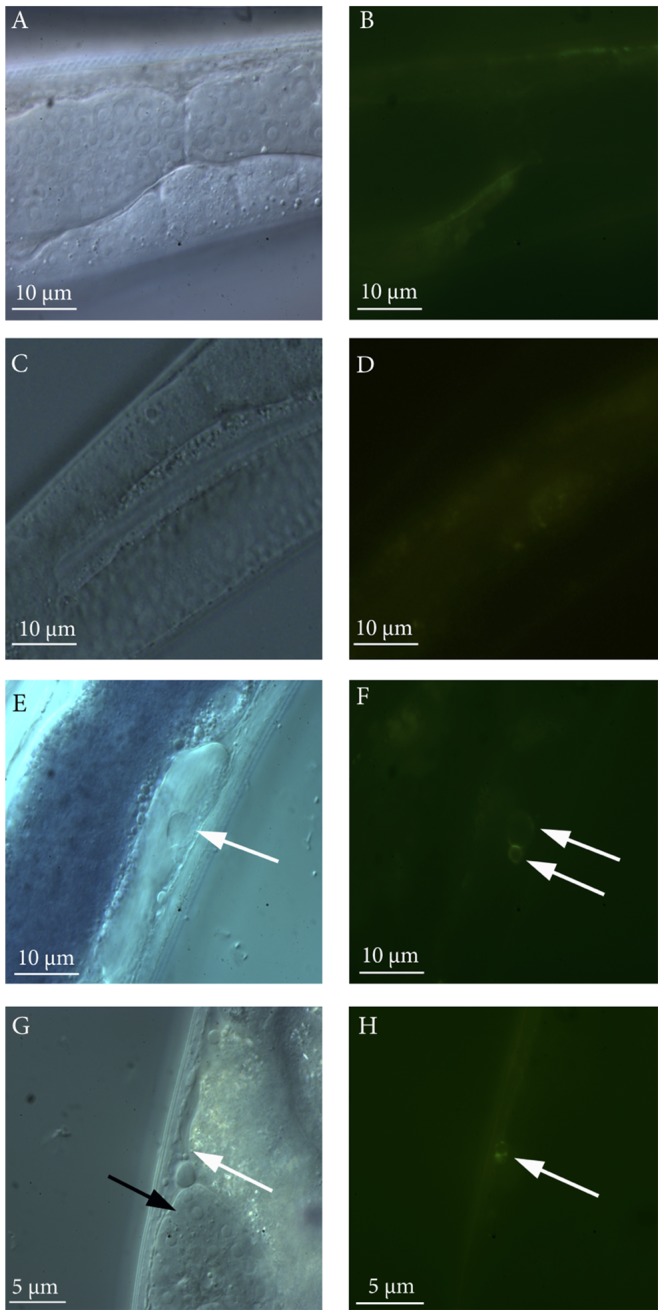
Apoptosis analysis in CED-1::GFP transgenic nematode strain CU1546. Visualization of GFP apoptosis marker inside the nematodes exposed to the violacein deficient mutant clones (20G8*vioA* ^−^ panels A and B; 20G8*vioC^−^* panels C and D) or violacein producing clone (20G8, panels E and F and G and H). Images show a section of the intestine and the gonads of *C. elegans* by DIC (panels A, C, E, G) and by epifluorescence microscopy (panels B, D, F, H). A ring-shaped GFP signal was detected in somatic cells of CU1546 nematodes exposed to 20G8 clone (two white arrows in panel F and white arrow in panel H) but not to 20G8*vioA* ^−^ and 20G8*vioC^−^* mutants (panel B and D). The ring-shaped GFP signal is associated with the expression of CED-1 receptor on the cell wall of engulfing cells only when phagocytosis of apoptotic cells is taking place. The ring-shaped GFP signal is apoptosis-specific and is visible in somatic cells of the nematodes (white arrows in E and G), the germ line (gonad) of the nematode is shown in panel G (black arrow). A non-specific fluorescence signal is visible in panels B and D.

To further elucidate the process of killing, transgenic nematodes with a GFP marker for apoptosis (CU1546 strain, Table 2) were exposed to a bacterial lawn of the violacein producing clone 20G8 and the violacein deficient mutant clones 20G8*vioA^−^* and 20G8*vioC* ^−^ for 24 hours, and thereafter visualised using differential interference contrast (DIC) and epifluorescence microscopy. The GFP signal was detected in somatic cells only in animals incubated with the 20G8 clone (70.3% of animals, n = 20), but not with 20G8*vioA^−^* and 20G8*vioC* ^−^ mutants (0%, n = 13 and n = 15 respectively) (Figure 4) showing that only the violacein-producing bacteria are capable of inducing apoptosis.

### Mutations in the IIS pathway influence the sensitivity of *C. elegans* to violacein-producing bacteria and pure violacein

**Figure 5:**
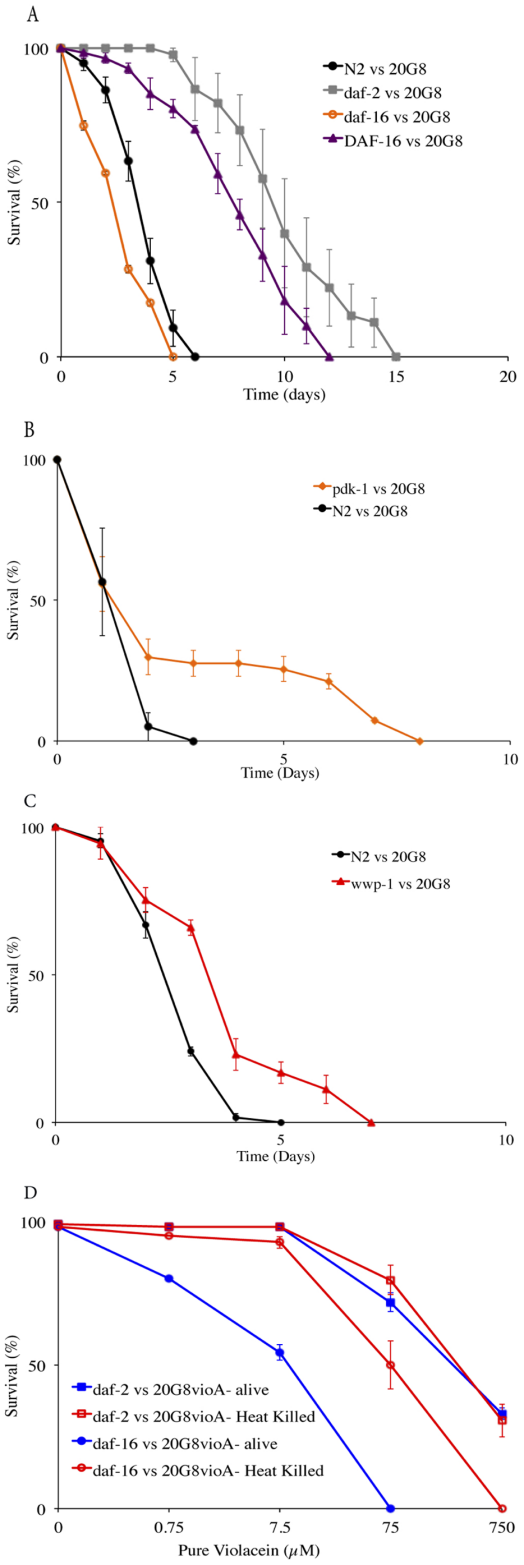
Nematode killing assay and dose response assay (wild type animals and *daf-2*, *daf-16, pdk-1, wwp-1* and DAF-16 mutant nematodes vs 20G8 clone). The survival of nematodes fed with 20G8 clone was measured for (A) *daf-2*, *daf-16* and DAF-16 mutant animals. (B) *pdk-1* mutant animals (C) *wwp-1* mutant animals (D) Dose response of *C. elegans daf-2* and *daf-16* strains to pure violacein added to 20G8*vioA^−^* mutant bacteria alive and heat killed. Each data point represents means ± the standard error of three replicate plates *p* values were calculated on the pooled data of all of the plates in each experiment by using a Students *t*-test on the number of surviving nematodes at day seven.

The IIS pathway with gene regulators DAF-2 and DAF-16 plays an important role in *C. elegans* pathogen defence [18]. We therefore questioned if *C. elegans* uses this pathway to also protect itself against violacein-mediated killing. To address this question, *C. elegans* loss of function mutants in the genes *daf-2* (CB1370), *pdk-1* (JT9609), *daf-16* (IU10) and *wwp-1* (RB1178), and a DAF-16 over-expressing strain (TJ356) (Table 2) were fed with the violacein-producing clone 20G8 in the nematode killing assay. The life span of *C. elegans* strains carrying loss of function mutations in *daf-2* and *pdk-1* and *wwp-1* were significantly increased compared to both wild type animals and *daf-16* mutant (*p*<0.0001) (Figure 5A, 5B and 5C). In contrast the *C. elegans* IU10 strain with loss of function mutation in the FOXO-family transcription factor DAF-16 displayed significantly reduced (*p*<0.0005) survival compared to wild type N2 animals (Figure 5A). Whereas viability of transgenic DAF-16::GFP nematodes that overexpress DAF-16 was significantly improved compared to both wild type and *daf-16* mutant animals (*p*<0.0001) (Figure 5A).

Similar results were observed for the *C. elegans* strains fed with the violacein deficient mutant (20G8*vioA* ^−^) supplemented with pure violacein in a dose response assay (Figure 5D). The toxic effect of violacein was dose dependent in wild type N2 nematodes and in *daf-2* and *daf-16* mutant animals (significant difference when nematodes of the same strain were exposed to 7.5, 75 or 750 µM of pure violacein, *p*<0.05). In these assays the lethal dose of violacein required to kill 50% of the population was higher for *daf-2* mutants (75 µM<LC_50_<750 µM) than for the wild type (7.5 µM<LC_50_<75 µM) or the *daf-16* mutant animals (7.5 µM<LC_50_<75 µM) (Figures 2B and 5D), further indicating that sensitivity or resistance to violacein is at least partially mediated by the IIS pathway.

### *C. elegans* daf-2 mutant and DAF-16 overexpressing strains are resistant to intestinal accumulation by the violacein-producing clone 20G8

Given that bacterial intestinal accumulation is in part responsible for the toxic phenotype of violacein producing cells and that the IIS pathway has previously been shown to influence bacterial accumulation in the nematode intestine [21], we aimed to determine if elements of the IIS pathway mediate resistance to violacein by preventing bacterial accumulation.

Firstly, we compared the sensitivity of various *C. elegans* strains to violacein when fed viable or heat killed bacteria. The presence of viable bacterial cells significantly increased the sensitivity of *daf-16* mutant animals to violacein compared to when the mutant was exposed to heat killed bacteria (significant difference when pure violacein was added to viable or heat killed bacteria *p*<0.05, Figure 5D). In contrast, heat killed bacteria had no or little impact on the survival of *daf-2* mutant (*p*>0.05, Figure 5D).

Secondly, we assessed the ability of *E. coli* clone 20G8 to accumulate in the intestine of the various *C. elegans* strains. Ninety one percent of *daf-16* mutant nematodes (n = 35) showed evidence of bacterial accumulation when exposed to 20G8 cells, in contrast no or little bacterial accumulation was present in the *daf-2*-loss of function and DAF-16 over-expressing mutants under the same treatment (0% n = 45 and 1.1% n = 31, respectively). Enlargement of extracellular regions was present in wild type and *daf-16* mutant nematodes (90% n = 20 and 92% n = 25, respectively) fed with clone 20G8 (arrowheads in Figure 3 panel A and panel D respectively) while *daf-2*-loss of function and DAF-16 over-expressing mutant animals showed normal anatomical structures and no tissue injury was detected (Figure 3 panel E 0% n = 19 and Figure 3 panel F 0% n = 16, respectively). Together these data indicate that the resistance to violacein by *daf-2* null mutants and DAF-16 over-expressing *C. elegans* strains is, in part, due to their improved ability to prevent accumulation of bacteria in the presence of violacein.

### Loss of function in IIS controlled genes results in increased sensitivity to violacein

**Figure 6:**
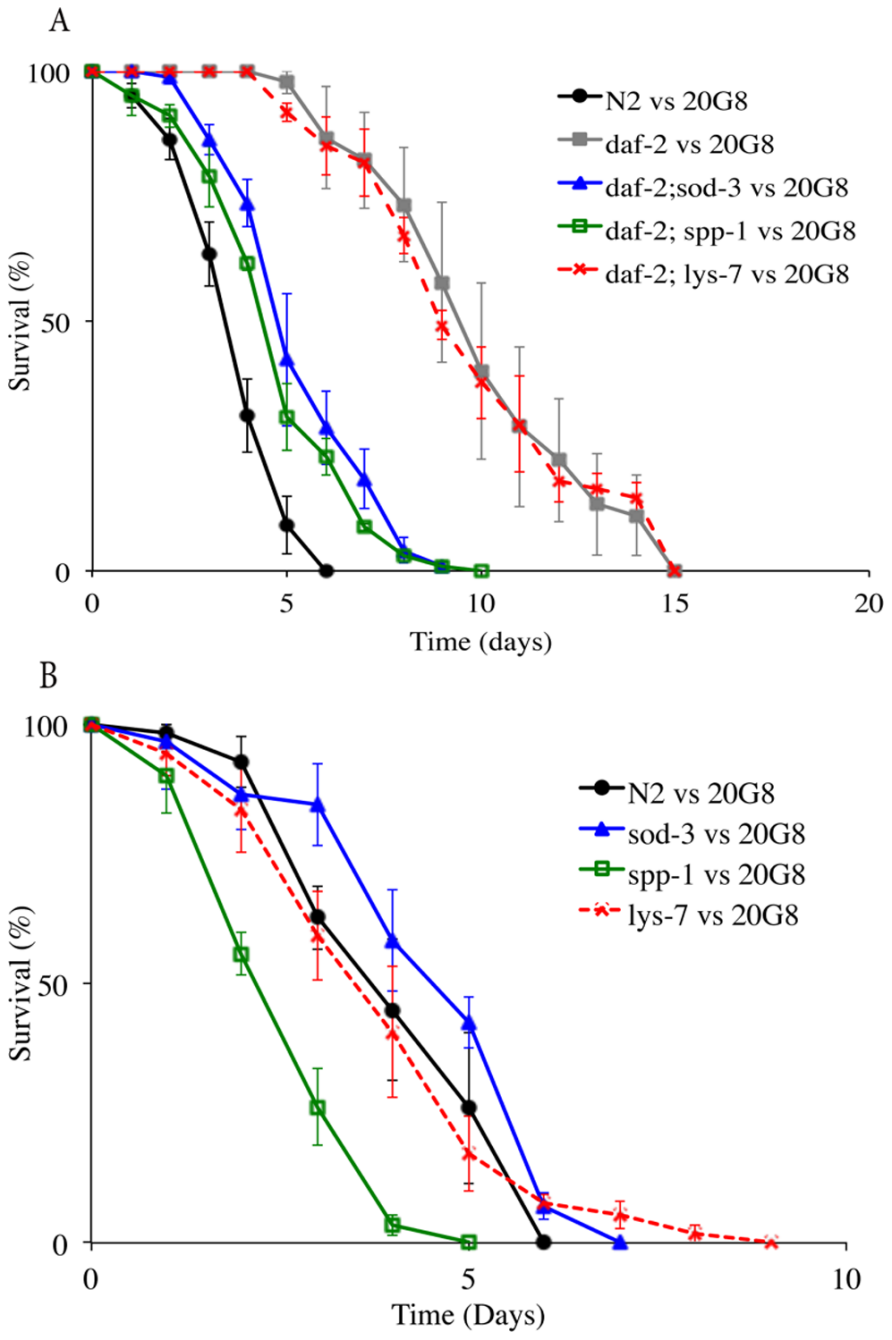
Nematode killing assay (wild type animals and *daf-2*, *daf-2;spp-1*, *daf-2;lys-7*, *daf-2;sod-3, spp-1, lys-7*, *sod-3* mutant nematodes vs the 20G8 clone). (A) The survival of the nematode was tested using *C. elegans* double mutants *daf-2;sod-3*, *daf-2;spp*-*1*, *daf-2;lys-7* and (B) the single mutant animals *sod-3*, *spp*-*1*, and *lys-7*. Each data point represents means ± the standard error of three replicate plates. *p* values were calculated on the pooled data of all of the plates done in each experiment by using the log-rank (Mantel–Cox) method and the values are provide in the text.

In *C. elegans* DAF2/DAF16 controls the expression of various effector genes including those relevant for detoxification and antimicrobial activity such as the superoxidase dismutase gene *sod-3* and antimicrobial genes *spp-1* and *lys-7* [23], [38]. Thus given that the precise molecular target/s for violacein in *C. elegans* are unknown we sought to determine which, if any, of these relevant downstream genes are required for the increased resistance to violacein observed in *daf-2* null and DAF-16 over-expressing strains. Specifically we chose to test violacein sensitivity in *C. elegans* mutants defective in *sod-3, spp-1* and *lys-7* (Table 2) because of the previous reported involvement of these genes in immunity to bacterial accumulation [39], [40], [41]. We found that *daf-2;spp*-*1* and *daf-2;sod-3* double mutants displayed significantly reduced survival compared to the single mutant *daf-2* (*p*<0.0001, Figure 6A) when exposed to the 20G8 clone in a nematode killing assay. No reduction in viability was detected in the *daf-2;lys-7* double mutant when compared to the single mutant *daf-2* (*p* = 0.937, Figure 6A). Interestingly a single mutation in gene *spp-1* significantly reduced the nematode's life span when compared to wild type animals (*p*<0.0001), while the viability of the nematode was not affected by mutations in the *lys-7* and *sod-3* genes (*p*>0.05, Figure 6B). These data indicate that resistance to violacein in *daf-2* mutants is at least in part driven by SPP-1 and SOD-3, with the antimicrobial LYS-7 having little or no involvement.

## Discussion

### Violacein has antinematode activity

In this study, we identified violacein as the metabolite responsible for the antinematode activity of *Microbulbifer* sp. D250. Violacein is arguably best known for its antibacterial properties and its activity as a potentially novel therapeutic against a range of tumors [16]. However, to the best of our knowledge, and with the exception of studies related to cancer therapy, this is the first report of violacein toxicity towards a multicellular eukaryote, thus adding to the list of biological functions for this natural metabolite.

Genetic analysis identified an operon of five conserved biosynthesis genes *vioA-E* that have been identified across all violacein-producing strains studied to date [7], [12], [42], [43]. Interestingly, mutations in *vioC* or *vioD* result in a grey colony pigmentation, reminiscent of the accumulation of the violacein precursor pro-violacein [7], [43], [44]. Pro-violacein differs from violacein by the absence of one oxo-group in the C15 position (indolyl instead of indolone, see Table 3 and reference [7]). This minor chemical difference, however, has a substantial impact on the toxicity, as the grey-pigmented *vioC* mutant did not kill nematodes in our study. Similar observation have also been recently made for *E. coli* K12 strains producing either violacein or pro-violacein, which were and were not resistant, respectively, to protozoan predation [45].

*C. elegans* is intrinsically more resistant to the effects of violacein (LC_50_>30 µM) than other bacterial grazers such as flagellates and amoebae, which were found to be effective at concentrations of 10 µM [46] and 1 µM [12] respectively. Nevertheless, violacein appears to be more potent towards *C. elegans* than toxins derived from known bacterial pathogens. For example, small phenazine molecules, including phenazine-1-carboxylic acid recently identified from *Pseudomonas aeruginosa*, are toxic to *C. elegans* only at concentrations greater than 70 µM. The effective concentration of violacein against *C. elegans* observed here is also similar to that of recent studies investigating novel antihelminthic therapies. For example, in a screen of existing drug leads Taylor *et al* [47] identified 18 candidate compounds having detectable phenotypes against *C. elegans* with EC_50_ ranging between 0.7 µM and >192 µM, including those already approved as cancer therapeutics such as Dasatinib (LC_50_ 22.3 µM) and Flavopiridol (LC_50_ 48.3 µM). Despite its many biological activities, the toxicity of violacein on (non-tumoral) mammalian cells is quite low. Recently, it has been reported that intraperitoneal doses of violacein of up to 1 mg kg^−1^ are not toxic to mouse blood, kidneys, or liver, thus enabling a potential *in vivo* use of violacein and its derivatives as a therapeutic compound with few side effects [48]. Given the relatively low effective concentration of violacein towards *C. elegans* determined here, we propose that the investigation of violacein as an antiparasitic compound is a reasonable prospect and that further studies regarding the activity of violacein against model parasitic nematodes, could reinforce the therapeutic potential of this drug.

### Bacterial accumulation in the intestine of *C. elegans* is a key factor involved in the toxicity of violacein

We observed that in the presence of violacein the otherwise non-pathogenic *E. coli* has the ability to accumulate in the intestine and eventually kill *C. elegans*. The exact mechanism by which violacein treatment leads to bacterial accumulation and reduced nematode viability is yet to be determined, however recent reports have demonstrated a link between nematode longevity and intestinal colonization [21]. Specifically, Portal-Celhay *et al* [21] showed that the capacity to control bacterial accumulation in the gut was dependent on the immunological status and age of the individual animal. Heavy bacterial accumulation has also been shown to reduce the lifespan of the nematodes depending on the bacterial strain used [21], [49]. Thus it is possible that exposure to violacein compromises the nematode's defence resulting in a reduced capacity to control bacteria in the gut and, thus, increasing the mortality rate. This is supported by similar observations recently made in various *Bacillus* species, in which treatment with the Bacillus pore-forming crystal protein (Cry PFP) seemingly sensitizes *C. elegans* to bacterial infection [50]. An alternative explanation is that the presence of violacein allows bacteria to penetrate the intestinal tissue resulting in a lethal infection. Whilst we did not observe bacteria within the tissue of nematodes exposed to violacein, previous reports have suggested internal infection as a possible cause of death in older nematodes [49], [51] and so this alternative possibility should not be dismissed.

### Violacein induces apoptosis in *C. elegans*

In addition to increased susceptibility to bacterial accumulation, violacein is capable of inducing apoptosis in *C. elegans* (Figure 4). Previous studies have revealed that apoptosis is also involved in violacein-mediated cell death in mammalian cell lines [16] and amoebae [12]. Thus although the exact molecular target of violacein in the eukaryotic cell is yet to be elucidated, the induction of an apoptosis-like cell death mechanism in multiple, distantly related eukaryotic systems (mammalian cells, amoeba and nematodes) suggest that an ancient, common eukaryotic cell process may be an additional target of violacein-driven toxicity.

### The insulin/IGF-1 signalling pathway contributes to the native defence against violacein

The innate immune response, including the IIS pathway with regulators DAF-2 and DAF-16, is a key component of the nematodes first line of defence particularly against pathogens [18], [19], [52]. Here we add to that knowledge by demonstrating that the IIS pathway also contributes to the nematodes native defence against secondary metabolites derived from non-pathogenic bacteria. The *C. elegans daf-2* and *pdk-1* deficient animals were significantly more resistant to the toxicity of violacein and the associated bacterial accumulation as compared to the wild type strain. These finding are consistent with the “long-lived” phenotype of the *C. elegans daf-2* mutant [53], which is also known to mediate the immune defence to bacterial infections [52], [54].

Since the molecular target of violacein-mediated toxicity in *C. elegans* remains to be elucidated the mechanisms involved in the nematode immune response towards violacein is unknown. However recent studies using *E. coli* expressing the *Pseudomonas aeruginosa* translational inhibitor exotoxin A (ToxA), have demonstrated that *C. elegans* induces an immune response towards ToxA, which the nematode detects indirectly via the toxin-mediated damage [55]. Others have also demonstrated activation of immunity and detoxification genes in response to damage to a variety of cellular functions [56], many of which could result from exposure to bacterial toxins. Thus such an effector-triggered immunity is likely to be widespread in animals and may function to enable bacteriovorus organisms such as *C. elegans* to discriminate between commensal and pathogenic bacteria [57]. Therefore once the molecular target of violacein is established it will be of interest to determine if *C. elegans* responds directly to the presence of violacein or rather to the associated inhibition of, or damage to, specific cellular functions.

Identifying genes under DAF-2/DAF-16 control that are involved in the increased resistance to violacein may provide further insight into the molecular/cellular target of this compound. Indeed assessment of violacein sensitivity of selected *C. elegans daf-2* double mutant strains in the current study indicates that while the antimicrobial lysozyme (LYS-7) is not involved in violacein resistance, both the superoxide dismutase SOD-3 and the antimicrobial peptide SPP-1 seem to play a role in host defence against violacein. The potential role of SOD-3 is consistent with previous findings that violacein can cause oxidative stress in human cancer cell lines [58]. However other studies have indicated that violacein acts as an antioxidant for the producing bacteria [59]. This apparent contradiction may result from the same molecule producing different responses depending on the target cells. *Spp-1* is expressed in the nematode intestinal cells and has previously been shown to be involved in the immune response against pathogens *Salmonella typhimurium* [41] and *P. aeruginosa* [60]. Results from this study support the idea that SPP-1 could also control the violacein induced intestinal accumulation of *E. coli* in *C. elegans* and further suggest a wider target spectrum for this peptide.

Despite the large body of knowledge surrounding the IIS pathway of the innate immune response in *C. elegans* and its role in stress resistance and pathogenesis, there is a paucity of information regarding its role in response to bacterial toxins. In one of the few studies to date, Chen *et al*. [22] demonstrated that the IIS pathway is involved in the cellular defence of *C. elegans* against *Bacillus thuringensis* crystal pore-forming toxins (Cry PFT). It appears likely that *C. elegans* uses this conserved pathway as a general bacterial toxin defence mechanism, as, similar to our findings for violacein, reduction in DAF-2 signalling confers resistance to Cry PFT, which is at least in part, dependent on DAF-16 function. However the newly described second branch of the IIS pathway mediated by WWP-1 appears to play a different role in defence against violacein, as in contrast to the hypersensitivity towards Cry PFT [22], *wwp-1* mutant animals were more resistant to violacein than wild type nematodes (Figure 5C). These contrasting observations may speak to differences in how the two toxins affect target cells and will be the subject of future studies investigating the cellular response of *C. elegans* to bacterial toxins.

## Conclusion

In their natural habitat nematodes, including *C. elegans*, are major predators for bacteria that, in turn, have evolved a number of predatory defence mechanism including the production of inhibitory metabolites [61]. This work presents, for the first time, violacein as an antinematode compound and assesses toxicity and the cellular response to violacein exposure in a *C. elegans* animal model. We found that this small molecule facilitates intestinal accumulation of the *E. coli* host cells and stimulates apoptotic activity. Mutations in a number of genes in the IIS pathway (*daf-2 and pdk-1*) and over-expression of another (*daf-16*) can confer significant resistance to violacein toxicity, providing evidence that *C. elegans* uses the DAF-2/DAF-16 innate immune signalling pathway to defend itself against this and potentially other noxious bacterial metabolites. Finally we demonstrate that defence against violacein requires, at least in part, the function of the antimicrobial peptide SPP-1 and possibly superoxide dismutase SOD-3. It is expected that the added knowledge will assist in the design of future mutational and/or gene expression studies aimed at determining the mechanisms of violacein toxicity in the nematode model including its direct molecular target/s. Such studies will not only prove important for further understanding the molecular basis for microbial antagonistic interactions but also for the development of microbial secondary metabolites such as violacein as novel chemotherapeutics.
