# Correction: Antinematode Activity of Violacein and the Role of the Insulin/IGF-1 Pathway in Controlling Violacein Sensitivity in *Caenorhabditis elegans*

**DOI:** 10.1371/journal.pone.0210026

**Published:** 2018-12-26

**Authors:** Francesco Ballestriero, Malak Daim, Anahit Penesyan, Jadranka Nappi, David Schleheck, Paolo Bazzicalupo, Elia Di Schiavi, Suhelen Egan

There is an error in the eleventh paragraph of the Results section. The correct paragraph is: In *C*. *elegans* DAF2/DAF16 controls the expression of various effector genes including those relevant for detoxification and antimicrobial activity such as the superoxidase dismutase gene *sod-3* and antimicrobial genes *spp-1* and *lys-7* [23,38]. Thus given that the precise molecular target/s for violacein in *C*. *elegans* are unknown we sought to determine which, if any, of these relevant downstream genes are required for the increased resistance to violacein observed in daf-2 null and DAF-16 over-expressing strains. Specifically we chose to test violacein sensitivity in *C*. *elegans* mutants defective in *sod-3*, *spp-1* and *lys-7* (Table 2) because of the previous reported involvement of these genes in immunity to bacterial accumulation [39,40,41]. We found that *daf-2;sod-3* double mutant displayed significantly reduced survival compared to the single mutant *daf-2* (p<0.0001, Fig 6A) when exposed to the 20G8 clone in a nematode killing assay. Interestingly a single mutation in gene spp-1 significantly reduced the nematode’s life span when compared to wild type animals (p<0.0001), while the viability of the nematode was not affected by mutations in the *lys-7* and *sod-3* genes (p>0.05, Fig 6B). These data indicate that resistance to violacein in *daf-2* mutants is at least in part driven by SPP-1 and SOD-3, with the antimicrobial LYS-7 having little or no involvement.

Fig 6 is incorrect. Please see the corrected Fig 6 here.

**Fig 6:**
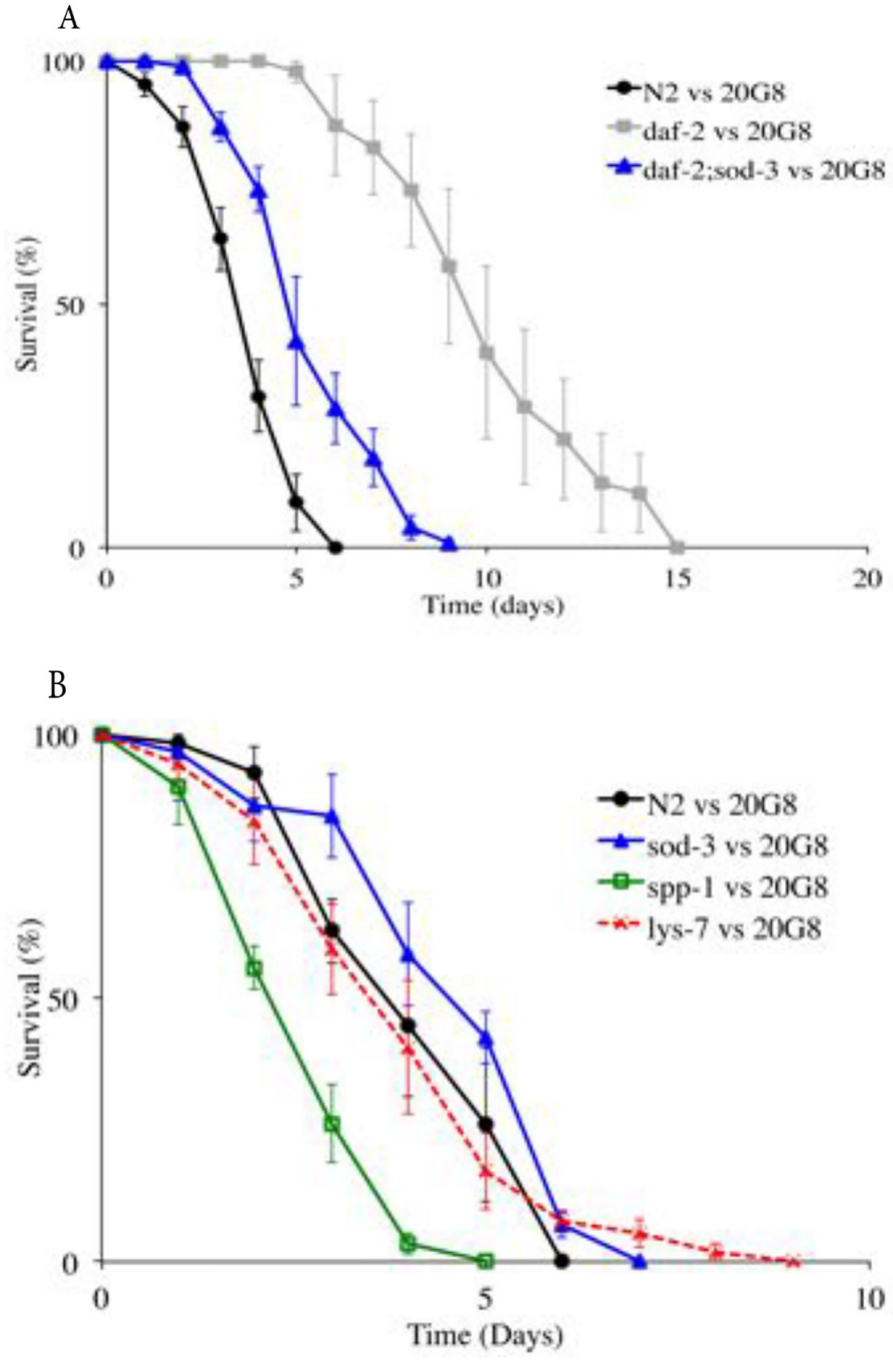
Nematode killing assay (wild type animals and *daf-2*, *daf-2;sod-3*, *spp-1*, *lys-7*, *sod-3* mutant nematodes vs the 20G8 clone). (A) The survival of the nematode was tested using *C*. *elegans* double mutant *daf-2;sod-3*, and (B) the single mutant animals *sod-3*, *spp-1*, and *lys-7*. Each data point represents means ± the standard error of three replicate plates. p values were calculated on the pooled data of all of the plates done in each experiment by using the log-rank (Mantel–Cox) method and the values are provided in the text.

Table 2 is incorrect. Please see the corrected Table 2 here.

**Table 2:** *C*. *elegans* strains used in this study.

Strain name	Genotype/allele designation	Relevant characteristics	Source or reference
N2 Bristol	*C*. *elegans* wild isolate	Wild type isolate	CGC^a^
CU1546	*smIs34*	ced-1p::ced-1::GFP + *rol-6(su1006)*	CGC^a^
CB1370	*daf-2*(*e1370*) III	Mutated in the insulin-like receptor DAF-2. Temperature sensitive dauer constitutive	CGC^a^
IU10	*daf-16*(*mgDf4*7) I; *rrf-3*(*pk1426*) II	Mutated in the FOXO-family transcription factor DAF-16	CGC^a^
TJ356	*zIs356* IV	Integrated DAF-16::GFP roller strain. Daf-c, Rol, fluorescent DAF-16::GFP. Overexpression of DAF-16	CGC^a^
JT9609	*pdk-1 (Sa680)* x	Mutation in the gene encoding for 3-phosphoinositide-dependent protein kinase	CGC^a^
RB1178	*wwp-1(ok1102) I*.	Mutation in the gene encoding for the WW domain protein 1	CGC^a^^b^
TM127	*daf-2*(*e1370*) III; *sod-3*(*sj134*) X	Double mutant in the insulin-like receptor DAF-2 and in the superoxide dismutase SOD-3	CGC^a^
GA186	*sod-3*(*tm760*) X	Mutated in the iron/manganese superoxide dismutase SOD-3	CGC^a^
RB1286	*lys-7*(*ok1384*) V	Mutated in the putative antimicrobial lysozyme LYS-7	CGC^a^
RB2045	*spp-1*(*ok2703*) III	Mutated in the antimicrobial peptide caenopore SPP-1	CGC^a^

^a^ *Caenorhabditis* Genetics Center, the University of Minnesota.

^b^ *C*. *elegans* Gene Knockout Project http://www.celeganskoconsortium.omrf.org.
